# Validation of the GALS musculoskeletal screening exam for use in primary care: a pilot study

**DOI:** 10.1186/1471-2474-9-115

**Published:** 2008-08-27

**Authors:** Karen A Beattie, Raja Bobba, Imaan Bayoumi, David Chan, Inge Schabort, Pauline Boulos, Walter Kean, Joyce Obeid, Ruth McCallum, George Ioannidis, Alexandra Papaioannou, Alfred Cividino

**Affiliations:** 1 Department of Medicine, McMaster University, Hamilton, ON, Canada; 2 Department of Kinesiology, McMaster University, Hamilton, ON, Canada

## Abstract

**Background:**

As the proportion of the Canadian population ≥65 grows, so too does the prevalence of musculoskeletal (MSK) conditions. Approximately 20% of visits to family physicians occur as a result of MSK complaints. The GALS (Gait, Arms, Legs, and Spine) screening examination was developed to assist in the detection of MSK abnormalities. Although MSK exams are primarily performed by rheumatologists or other MSK specialists, expanding their use in primary health care may improve the detection of MSK conditions allowing for earlier treatment. The primary goal of this study was to evaluate the use of the GALS locomotor screen in primary care by comparing the results of assessments of family physicians with those of rheumatologists. The secondary goal was to examine the incidence of MSK disorders and assess the frequency with which new diagnoses not previously documented in patients' charts were identified.

**Methods:**

Patients ≥65 years old recruited from an academic family health centre were examined by a rheumatologist and a family physician who recorded the appearance of each participant's gait and the appearance and movement of the arms, legs and spine by deeming them normal or abnormal. GALS scores were compared between physicians with the proportion of observed (P_obs_), positive (P_pos_) and negative (P_neg_) agreement being the primary outcomes. Kappa statistics were also calculated. Descriptive statistics were used to describe the number of "new" diagnoses by comparing rheumatologists' findings with each patient's family practice chart.

**Results:**

A total of 99 patients consented to participate (92 with previously diagnosed MSK conditions). Results showed reasonable agreement between family physicians and rheumatologists; P_obs _= 0.698, P_pos _= 0.614 and P_neg _= 0.752. The coefficient of agreement (estimated Kappa) was 0.3675 for the composite GALS score. For individual components of the GALS exam, the highest agreement between family physicians and rheumatologists was in the assessment of gait and arm movement.

**Conclusion:**

Previously reported increases in undiagnosed signs and symptoms of musculoskeletal conditions have highlighted the need for a simple yet sensitive screening exam for the identification of musculoskeletal abnormalities. Results of this study suggest that family physicians can efficiently use the GALS examination in the assessment of populations with a high proportion of musculoskeletal issues.

## Background

Musculoskeletal conditions are commonly seen in health care practices and have been identified as the leading cause of chronic health problems, long term disability and consultations with health professionals in Canada [1-3]. Given the fact that many of these conditions are associated with aging (i.e. osteoporosis, osteoarthritis), this burden on society is estimated to increase in the future as the proportion of elderly individuals increases [4]. A 2007 Statistics Canada report revealed that 13.7% of Canadians are 65 years of age or older with the fastest growing group being those between 55–64 years of age [5]. In 1998, it was estimated that musculoskeletal issues accounted for approximately 20% of the daily care administered by family practitioners [6]. However, it is also known that signs and symptoms of musculoskeletal conditions may be under diagnosed by primary care physicians as it has been suggested that the examination of this system is often omitted from routine patient assessments [6,7]. This may be related to the notion that some primary care physicians may not feel that, for the most part, they can alter the clinical course by early detection, such as may be the case in osteoarthritis. Studies of medical students and practicing physicians have cited a lack of interest and/or a lack of perceived importance of the musculoskeletal system, time constraints and an overall lack of confidence in providing assessment as reasons for the discomfort with managing patient musculoskeletal conditions [6,8-12].

Given the gap between the incidence and diagnosis of musculoskeletal diseases in the primary care population, a simple screening exam may enable practitioners to accurately identify abnormalities of this system. The GALS locomotor screening exam, an acronym that stands for Gait, Arms, Legs and Spine, has been developed by Doherty and colleagues in response to this need [4]. This 3-minute examination consists of three questions about pain, difficulty dressing and difficulty with stairs, followed by assessment of the appearance and movement of the four regions. Although it cannot be considered a substitute for a more detailed locomotor exam, it may be useful as a diagnostic tool for the identification of musculoskeletal abnormalities and possible subsequent early intervention.

To date, the GALS exam is primarily carried out by rheumatologists or musculoskeletal specialists as a teaching tool [9,13]. However, its introduction to the medical school curriculum in Britain and recently in Canada has proven to be beneficial. Medical students taught the GALS examination reported feeling more confident when assessing the locomotor system and, when evaluated by rheumatology consultants in an examination, performed the screen with the same degree of skill as other clinical areas (i.e. chest examination, blood pressure) [13,14]. The GALS screening exam is ideally suited for the family physician who has the opportunity, as a first contact in the health care system, to identify musculoskeletal disorders since it can be easily incorporated into a routine physical exam. However, while the examination is proven to be both valid and reliable when conducted by specialists in rheumatology, its effectiveness in the primary care setting has not yet been determined [7,15]. The aims of this pilot study were 1) to evaluate the accuracy of the GALS examination by primary care physicians as compared to rheumatologists; 2) to test a sampling frame method for participant recruitment and 3) to determine if the instructional DVD is sufficient in teaching the GALS exam.

## Methods

### Participants

Potential study participants from a local academic family practice clinic were selected through a database generated from the electronic medical record, Open Source Clinical Applications Resource (OSCAR) [16]. This is a large family practice centre which is representative of the general population. All patients 65 years of age and older who were capable of giving informed consent were considered eligible to participate, regardless of their medical history. International Statistical Classification of Diseases and Related Health Problems (ICD-9) codes were known for each potential study participant in the OSCAR database. The intention was to select approximately half of the subject population with no record of ICD-9 code between 710 and 739 (Diseases of the Musculoskeletal System and Connective Tissue) with the other half having such a record. A list of approximately 261 eligible patients was generated by the database manager and given to the study co-coordinator who was responsible for mailing information letters to all eligible participants. The first 50 patients with no known musculoskeletal disorders and the first 50 patients with known musculoskeletal disorders who positively responded to a follow-up telephone call inviting them to participate were to comprise the study population. Those willing to take part in the study were then scheduled for a one hour appointment on one of three study days.

To estimate sample size we attempted to estimate the reliability coefficient with as much accuracy as possible to be certain that the true reliability coefficient was reasonably close to the estimate. We hypothesized that an intraclass correlation coefficient (ICC) between the two groups of physicians (family physicians and rheumatologists) was approximately 0.7. Based on the equation derived from Bonett, we determined that 100 subjects would be needed for an estimated ICC of 0.7 and precision ± 0.10 [17]. Thus, recruitment was closed once the target convenience sample of 100 subjects was achieved. This study was approved by the Research Ethics Board at Hamilton Health Sciences and McMaster University.

### Physician Participation

Four rheumatologists (AC, RB, PB, WK) agreed to participate in this validation study, all of whom had previous experience using the GALS exam in routine clinical practice. Three family physicians (IB, DC, IS), all members of the Canadian College of Family Physicians (CCFP), from the Stonechurch Family Health Centre also volunteered to participate in the study. Participating family physicians had never been previously exposed to or received training on the GALS examination. An instructional DVD of the GALS exam, endorsed by the Canadian Rheumatology Association, was used as the primary teaching method and was distributed to each family physician 2 months prior to conducting the study. This DVD, which takes approximately five minutes to review, demonstrates a rheumatologist performing the exam, as well as 3 case studies on adult patients with specific abnormalities. All of the family physicians received a call one week prior to the scheduled physical exam date to clarify any questions regarding the exam. No additional training was provided.

### Study Procedures

Each study participant was assessed by one family physician and one rheumatologist, both of whom were blinded to the medical history of the patient. Family physicians and rheumatologists examined the patients immediately following one another on the same exam day. During the examination, physicians posed three questions to each participant and then proceeded to score each of the 7 components of the GALS examination as being either abnormal or normal as shown in Table 1. Details about what features of each of the GALS components were examined and assessed to yield a normal or abnormal appearance or movement are shown in Table 2. All examiners were blinded as to the assessments of the other physicians. Family physicians were asked to check the appropriate box in the record form (abnormality: yes or no) and document the observed abnormalities. They were not required to make a diagnosis. Rheumatologists, on the other hand, were also asked to state the presence of absence of abnormalities and, in the case where an abnormality was identified, perform a focused exam to assess the abnormality and make a diagnosis, if possible.

**Table 1:** GALS Recording Sheet Completed by Physicians

	**Yes**	**No**
**Do you have any pain or stiffness in your muscles, joints or back?**		
**Do you have any difficulty dressing yourself completely?**		
**Do you have difficulty walking up or down stairs?**		
**Gait**	Abnormal or Normal	
	**Appearance (✔ or ✘)**	**Movement (✔ or ✘)**
**Arms**		
**Legs**		
**Spine**		

✔ = normal, ✘ = abnormal

**Table 2:** Individual features of the GALS exam which were examined (6)

**GAIT**
• Symmetry & smoothness of movement
• Stride length & mechanics
• Ability to turn normally & quickly
**ARMS (Hands)**
• Wrist/finger swelling/deformity
• Squeeze across 2^nd ^to 5^th ^metacarpals for tenderness (indicates synovitis)
• Turn hands over, inspect muscle wasting & forearm pronation/supination

**ARMS (Grip Strength)**
• Power grip (tight fist)
• Precision grip (oppose each finger to thumb)

**ARMS (Elbows)**
• Full extension

**ARMS (Shoulders)**
• Abduction & external rotation of shoulders

**LEGS (Feet)**
• Squeeze across metatarsals for tenderness (indicates synovitis)
• Calluses

**LEGS (Knees)**
• Knee swelling/deformity, effusion
• Quadriceps muscle bulk
• Crepitus during passive knee flexion

**LEGS (Hips)**
• Check internal rotation of hips

**SPINE (Inspection from behind)**
• Shoulders & iliac crest height symmetry
• Scoliosis
• Paraspinal, shoulder, buttocks, thighs & calves muscles normal
• Popliteal or hind foot swelling or deformity

**SPINE (Inspection from front)**
• Quadriceps normal in bulk & symmetry
• Swelling or at Varus or valgus deformity at knee
• Forefoot of midfoot deformity, action normal
• Ear against shoulder on either side to check lateral cervical spine flexion
• Hands behind head with elbows back (check rotator cuff muscles, acromioclavicular, sternoclavicular & elbow joints)

**SPINE (Inspection from side)**
• Normal thoracic & lumbar lordosis
• Normal cervical kyphosis
• Normal flexion (lumbosacral rhythm from lumbar lordosis to kyphosis) while touching toes

**SPINE (Trigger point tenderness)**
• Supraspinatus muscle tenderness (exaggerated response)

Analyses were performed to assess the degree of overall agreement (P_observed_), as well as the degree of agreement on traits considered to be abnormal (P_positive_) and normal (P_negative_) between the family physicians' and rheumatologists' scores on the GALS assessment. Kappa statistics and 95% confidence intervals (95% CI) were calculated as a composite of the overall GALS examination as well as for each component of the exam (Gait, Arms, Legs, and Spine).

In order to assess the usefulness of the GALS examination in identifying abnormalities not previously detected in routine family practice, electronic charts (e-charts) including family physician and radiographic reports and referrals to physical therapy and tertiary care (i.e. rheumatologists), were retrospectively reviewed from the family practice clinic. Each chart was evaluated by two independent individuals. Physical abnormalities identified by the rheumatologists were sought in the chart history during the preceding 2 years; those that had not been previously documented were considered "new" abnormalities/diagnoses. Due to the difficulty in differentiating between acute and chronic pain during the examination, pain noted by rheumatologists on the GALS assessment form but not in the patient chart was not considered to be a new abnormality. In addition, all abnormalities recorded during the GALS examinations were reviewed by two rheumatologists (AC, RB). Those that could be investigated in further detail or treated, but not found in the patient charts, were considered to be previously undetected. All newly detected abnormalities were then grouped by trait, and further subdivided into affected region including: gait (antalgic, abnormal stride length), arms (fingers and hand, wrist, elbow, shoulder), legs (toes and feet, ankle, knee, hip, other), and spine (scoliosis, lordosis, kyphosis, decreased range of motion, other). Analyses were performed to assess the total number of abnormalities in each region were considered to be new as compared to those that were not, as well as the relative contribution of new abnormalities from each of gait, arms, legs and spine to the total. Data were analyzed using SPSS 12.0 for Windows XP Professional (SPSS Inc; Chicago, IL).

## Results

Of 261 individuals who were mailed a letter informing them about the study, 221 were reached by phone and invited to participate. Unfortunately however, the database-generated list of eligible patients included very few potential participants with no previous musculoskeletal diagnosis (ICD-9 codes 710–739) thus supporting the evidence of high prevalence of MSK disorders in those over 65 years of age. Regardless of ICD-9 code selection, those who responded positively (N = 103) were scheduled to be seen on one of three study days. Of those 103 scheduled for examination, 99 individuals were seen in the clinic, 92 of whom were identified as having a previously diagnosed musculoskeletal condition. Two individuals who were scheduled but not seen were sick on the exam day while two others did not show for their appointments. Those who did not agree to participate gave various reasons for declining, including illness and unavailability on study days. Of those who consented to participate, 61 (62%) were women and 38 were men with a group mean age of 75.2 (SD = 6.1) years (min. to max.: 65 to 89 years). Of the 99 participants, 7 were assessed by their own family physician (7%) by chance alone. Because the names of study participants' family physicians were not known at the time the study was designed and conducted, this occurred purely by chance. However, it should be noted that family physicians had no advance knowledge of the names of the patients they would be assessing and had no access to the patients' medical charts at any time before, during or after the study.

Overall, the observed agreement (P_obs_) of the GALS examination was 0.698 with a P_pos _of 0.614 and a P_neg _of 0.752. The composite GALS score had a coefficient of agreement (estimated Kappa) of 0.3675 (95% CI: 0.3009, 0.4342). Agreement was further subdivided into each component of the GALS exam as outlined in Table 1. The number of normal and abnormal features graded by each of the family practitioners and rheumatologists are displayed in Table 3. Comparisons between family physicians and rheumatologists are presented in Table 4. As shown in the table, agreement between physicians was highest in response to the three questions posed. It is intuitive that, when asked the same question by two different physicians, the patient would answer the same way the vast majority of the time since there is no bias or interpretation introduced by the physician.

**Table 3:** Number of features graded normal and abnormal for each patient

	**Primary Care Physician**		**Rheumatologist**	
	
	**Normal (N)**	**Abnormal (N)**	**Normal (N)**	**Abnormal (N)**
Arms – Appearance	66	33	62	36*
Arms – Movement	62	36*	67	30*
Legs – Appearance	53	43*	45	53*
Legs – Movement	43	54*	47	52
Spine – Appearance	73	25*	57	41*
Spine – Movement	62	36*	52	45*

*Please note that these features contain missing data (i.e. N(normal) + N(abnormal) ≠ 99).

**Table 4:** Agreement between Family Physician & Rheumatologist GALS Scores

	**P_POS_**	**P_NEG_**	**P_OBS_**	**Estimated Kappa (95% CI)**
**Pain/Stiffness**	0.944	0.758	0.910	0.704 (0.500, 0.908)
**Difficulty Dressing**	0.731	0.904	0.858	0.636 (0.444, 0.832)
**Difficulty on Stairs**	0.909	0.911	0.910	0.821 (0.694, 0.947)
**Gait**	0.676	0.813	0.784	0.490 (0.310, 0.670)
Arms – Appearance	0.617	0.793	0.742	0.412 (0.222, 0.601)
Arms – Movement	0.634	0.821	0.760	0.458 (0.271, 0.646)
Legs – Appearance	0.574	0.583	0.578	0.164 (0.000, 0.359)
Legs – Movement	0.711	0.666	0.690	0.379 (0.196, 0.563)
Spine – Appearance	0.400	0.697	0.597	0.128 (0.000, 0.314)
Spine – Movement	0.632	0.743	0.697	0.385 (0.204, 0.566)

Electronic charts for retrospective review were available for 92 of the 99 participants. Ten (10%) participants were identified with a new gait abnormality, nine requiring further investigation/referral. One-third of participants (N = 30) were identified with ≥1 new arm abnormality, the majority (N = 84%) being in the fingers/hand. Of 31 arm abnormalities, 7 would require further investigation or referral and 23 would be treated if symptomatic. In the legs, 35 (38%) participants had 46 new abnormalities, 18 requiring further investigation and 24 requiring treatment if symptomatic. Of these, 52% (N = 24) were in the toes/foot, 7% (N = 3) in the ankle, 24% (N = 11) in the knee, 15% (N = 7) in the hip and 2% (N = 1) "other". The prevalence of new spinal abnormalities was 29% (N = 27), with a total of 40 identified, 14 of which would require further investigation/referral. Scoliosis accounted for 15% of abnormalities (N = 6), kyphosis for 30% (N = 12), loss of lordosis for 20% (N = 8), decreased cervical ROM for 23% (N = 9), DDD for 10% (N = 4) and "other" for 3% (N = 1). These results are shown in Table 5.

**Table 5:** Prevalence of newly detected abnormalities by subcategory

Subcategory	Prevalence [% in category (% overall)]
**Gait**	

Stride Length	90.0 (7.2)
Antalgic	10.0 (0.8)

**Arms**	

Fingers/hand	83.9 (20.8)
Wrist	3.2 (0.8)
Elbow	3.2 (0.8)
Shoulder	9.7 (2.4)

**Legs**	

Toes/foot	52.2 (19.2)
Ankle	6.5 (2.4)
Knee	23.9 (8.8)
Hip	15.2 (5.6)
Other	2.2 (0.8)

**Spine**	

Scoliosis	15.8 (4.8)
Kyphosis	26.3 (8.0)
Loss of lumbar lordosis	21.1 (6.4)
Decreased cervical ROM	31.6 (9.6)
Degenerative disk disease	2.6 (0.8)
Other	2.6 (0.8)

## Discussion

While a few studies have investigated the reliability, sensitivity, and specificity of the GALS examination [7,15,18,19], we believe this to be the first study to investigate its use in primary care by comparing the results of the GALS exam between family physicians and rheumatologists. Results of this pilot study revealed a reasonable level of agreement between rheumatologists and family physicians recently taught to perform the GALS examination via an instructional DVD (estimated Kappa = 0.3675; 95% CI: 0.3009, 0.4342, P_obs _= 0.698). Upon further analysis of the individual components of the exam, assessments of gait and arm movement were found to have the greatest level of agreement, while the appearance of the legs and spine were identified as the sources of greatest disagreement. Gait is an extremely important component of the GALS exam since its assessment often contributes information with respect to a patients' propensity to falling [20,21,21].

To more accurately assess the source of disagreement, positive and negative agreement of all components of GALS were determined. Results revealed that family physicians were more likely to agree with rheumatologists when the trait being assessed was considered normal as opposed to abnormal, as shown in both Tables 3 and 4. Similarly, Hood and colleagues reported greater negative predictive values in the assessment of 200 patients suffering from acute or chronic musculoskeletal conditions, also suggesting that negative or normal traits are more easily identified [18].

Despite the fact that few studies have examined the use of GALS by different health care professionals, a pattern that has previously emerged, and one that was also noted in the current study, is the variation in the assessment of the appearance of the spine. Plant et al. investigated the reliability of the GALS examination when conducted by senior house officers and registrars in rheumatology (N = 30) and reported the greatest disagreement when scoring of the appearance of the spine [7]. Jones et al. also reported difficulties in the identification of other spinal abnormalities, particularly for lateral cervical flexion [19]. It has been suggested that age-related changes affecting flexibility of the neck and back are the likely source of the difficulties encountered in differentiating normal from abnormal spinal appearance [7,19]. Thus, it is plausible that these and other age-related changes may also contribute to difficulties distinguishing normal from mildly abnormal traits in other components of the GALS examination.

Further comparisons of our results with those of Plant et al. revealed a similar level of observed agreement; however, the reported reliability (estimated kappa) differed significantly. A well-known and frequently observed trend is that of the relation between reliability and degree of scale complexity (i.e. dichotomous scales, Likert scales etc.) where an increase in the number of possible outcomes (i.e. none, mild, moderate, or severe) results in increased reliability [22]. The decreased level of reliability as assessed by the kappa statistics (min = 0.13, max = 0.49) in this study may, in part, be attributable to the dichotomous nature of the scale employed where appearance and movement could be labeled only as normal or abnormal. In contrast, Plant et al. replaced the traditional dichotomous scale with one that allowed examiners to rate features as normal, mildly, moderately, or severely abnormal and subsequently reported higher kappa statistics varying from 0.49 to 0.74 [7].

Although the results of the current study appear to suggest that difficulties persist in the recognition of musculoskeletal abnormalities, one should be cautioned about making definitive conclusions without acknowledging factors which may have contributed to or limited the observed level of agreement. For instance, a review of patient scoring sheets completed by family physicians and rheumatologists revealed that while both examiners recognized similar patient characteristics, there was discrepancy between the comments recorded and the identification of these features as normal or abnormal. In a given patient, for example, some physicians recorded gait to be abnormal due to an observed limp, while others also noted the presence of a limp but incorrectly labeled this as normal. This observation helps to explain the trend observed in other features of the GALS exam where rheumatologists consistently labeled more features as abnormal than family practitioners as seen in Table 3. This may be another example of what some physicians may deem normal, age-related changes, thus assessing the feature as normal, while others would assess the feature as being abnormal relative to a healthy standard. This discrepancy may be linked to differences in the perception of abnormalities between family physicians and rheumatologists. However, given the fact that the recorded observations could not be objectively quantified or assessed as being mildly or moderately abnormal as in the study by Plant et al., these differences ultimately resulted in a decreased level of agreement. It is believed that agreement would have improved significantly had the newly trained family physicians been given an opportunity to directly observe the GALS examination as conducted by a rheumatologist and to meet with rheumatologists prior to the study to discuss characteristics that differentiate normal features from those that are abnormal. By coming to a consensus as to how to score certain features (i.e. the limp), it is anticipated that agreement would have been higher. In addition, variation in scoring between family physicians and between rheumatologists was not assessed. Characteristics of the cohort can also influence the measures of agreement, particularly the kappa statistic. For instance, the lack of symmetry in the study population (i.e. the majority have a musculoskeletal condition) will tend to produce lower kappa values [23,24]. One of the major limitations to this study was the asymmetry in the study population which consisted of only 7 participants who had never been identified with any musculoskeletal conditions by the ICD codes.

The prevalence of MSK abnormalities in this ambulatory study population was also estimated for each anatomical region. These were further subdivided by the joints that were involved. It was apparent that the most common features assessed as being abnormal by the rheumatologists were those in the joints of the fingers/hands (20.8% of patients) and the toes/feet (19.2% of patients). The vast majority of these abnormalities were cases of osteoarthritis in the peripheral joints, none of which had previously been documented in the patients' family practice charts. Decreased cervical range of motion (9.6% of patients) and abnormal knees (8.8%) were also prevalent in this population and were regions that had not been documented as abnormal by the patients' family physicians. There may be a couple of reasons for these "new" abnormalities; a) lack of documentation by the family physician, b) the patient has experienced these problems but not expressed/reported them to his/her family physician. The majority of these newly identified abnormalities would require further investigation (i.e. kyphotic posture being assessed for osteoporosis) or treatment (swollen joint treated with medication). Only one other study has used the GALS exam to investigate the prevalence of MSK abnormalities. This study was conducted in acute and chronic medical in-patients [18]. Here the GALS screening tool was positive (abnormality identified) in 53% of acute patients and 94% of chronic patients where osteoarthritis accounted for the majority of rheumatological conditions identified in the both study populations.

A future study will include a wider variation in subject ages so as to obtain a sample population without any previous musculoskeletal diagnoses allowing the sensitivity and specificity of the GALS exam to be investigated. This study will also involve the analyses of subgroup of patients who are assessed by all family physicians and all rheumatologists to assess the inter-observer variation. In addition, these results also suggest that an instructional DVD alone may not be the most effective and consistent method of teaching the GALS exam but that the DVD should be accompanied by oral instruction/interaction. This may be more important when instructing physicians who have already developed their skill set or routine as compared to medical students who have little to no background in this area.

Although the ability of family physicians to assess the MSK system prior to the introduction of the GALS examination was not assessed, our results suggest that family physicians can efficiently use the GALS examination to assess the MSK system, by integrating it into their routine physical exam. Nevertheless, previous studies of medical professionals whose ability to assess the MSK system before versus after learning the GALS examination was evaluated have revealed that physicians' confidence and efficiency in examining the system had increased significantly [13,14]. These results suggest that the same may be true for the family physicians of the current study.

## Conclusion

A report from the Summit on Standards for Arthritis Prevention and Care, a large multidisciplinary group including health care professionals and patients, clearly identified a need for a screening exam that could facilitate the identification of musculoskeletal diseases [25]. The results of this study support the notion that the GALS examination is a simple, reliable, and valid screening tool that may improve recognition of musculoskeletal abnormalities when used by specialists and family physicians alike. However, it also appears as though further validation needs should be considered by investigating different methods of training and scoring in a more diverse study population. This will be the focus of a future larger study. Although it cannot serve as a replacement for a focused rheumatological exam, it is believed that the use of the GALS exam in primary care settings may lead to increased detection of previously unrecognized abnormalities and early intervention in hopes of preventing further deterioration. For example, the early diagnosis of rheumatoid arthritis is associated with better long term outcome [26]. While detection of musculoskeletal conditions has significantly improved over the last decade, additional emphasis must be placed on educating primary care physicians to differentiate between normal age-related changes of the musculoskeletal system and early signs of deterioration that are mildly abnormal in nature as these are typically overlooked.

## Competing interests

The authors declare that they have no competing interests.

## Authors' contributions

KAB, AC, AP, DC and RB were responsible for the development and design of the study. AC, RB, PB and KW were the rheumatologists involved in the assessment of all patients. IB, DC, and IS were the family physicians involved in the assessment of all patients. GI was charged with all statistical analyses. KAB, JO and RMcC were involved in participant recruitment and organization of the study. All authors were involved in the writing of the manuscript. All authors read and approved the final manuscript.

## Pre-publication history

The pre-publication history for this paper can be accessed here:


